# Respiratory Pathophysiology of Mechanically Ventilated COVID-19 Patients

**DOI:** 10.7759/cureus.20218

**Published:** 2021-12-06

**Authors:** Anas Khalil, Atif Aljohani, Bashayer Alemam, Fardus Alshangiti, Fatmah A Jeddo, Hayam Albadi, Hind M Alshanqiti, Raghad Almughazzawi

**Affiliations:** 1 Internal Medicine, Taibah University, Al-Madinah Al-Munawarah, SAU; 2 Internal Medicine, Ohoud Hospital, Al-Madinah Al-Munawarah, SAU; 3 Medicine, Taibah University, Al-Madinah Al-Munawarah, SAU

**Keywords:** static compliance, peak inspiratory pressure, plateau pressure, partial pressure of arterial oxygen/ fractional inspired oxygen, respiratory pathophysiology, positive end-expiratory pressure, mechanical ventilation, severe acute respiratory syndrome coronavirus-2, coronavirus disease 2019, acute respiratory distress syndrome

## Abstract

Background and objectives

Coronavirus disease 2019 (COVID-19) is mainly a disease of the respiratory system that can lead to acute respiratory distress syndrome (ARDS). The pathophysiology of COVID-19 ARDS and consequently its management is a disputable subject. Early COVID-19 investigators hypothesized that the pathogenesis of COVID-19 ARDS is different from the usual ARDS. The aim of this study was to describe the lung mechanics in mechanically ventilated COVID-19 patients with ARDS.

Methodology

An observational retrospective cohort study was conducted on adult COVID-19 patients with ARDS who needed mechanical ventilation in the ICU of Ohoud Hospital, Madinah, KSA, from June to September 2020. Data were collected from the patients’ medical charts and electronic medical records and analyzed using Statistical Package for the Social Sciences (SPSS) software package version 22 (IBM Corp., Armonk, NY) for descriptive statistical analysis.

Measurements and main results

A total of 52 patients were analyzed: on intubation, the median positive end-expiratory pressure (PEEP) was 10 cm H_2_O (IQR, 2.3-16), the median plateau pressure was 27 cm H_2_O (IQR, 12-40), and the median driving pressure was 17 cm H_2_O (IQR, 3-30). The median static compliance of the respiratory system was 24.7 mL/cm H_2_O (IQR, 12.8-153.3). 59.5% had severe ARDS (the PaO_2_/FiO_2_ ratio was less than 100 mmHg), and 33% had moderate ARDS (the PaO_2_/FiO_2_ ratio ranged from 100 to 200 mmHg).

Conclusion

Our results suggest that the lung mechanics in COVID-19 ARDS patients who need mechanical ventilation do not differ from non-COVID-19 patients.

## Introduction

In late December 2019, an outbreak of severe acute respiratory syndrome coronavirus-2 (SARS-CoV-2) emerged that was later named coronavirus disease 2019 (COVID-19) [1,2]. About 2.6 million patients have died from the disease with a case fatality rate (CFR) of 2.2% [3]. COVID-19 has not only caused significant loss of human life but also poses an unprecedented challenge to global economy, poverty and public health [4].

COVID-19 is mainly a disease of the respiratory system resulting in pneumonia and then acute respiratory distress syndrome (ARDS) [1,2,5]. ARDS is an acute lung inflammation that affects both lungs and cause leakage of plasma and blood into the alveoli, leading to non-cardiogenic pulmonary edema. Subsequently, this causes shunt-related hypoxemia, low lung compliance and reduced ventilatable lung parenchyma [6,7]. Hence, the treatment of ARDS is focused on improving oxygenation, preventing further lung injury and increasing lung homogeneity [6]. This can be achieved by lung recruitment using high positive end-expiratory pressure (PEEP) and prone positioning, low tidal volume ventilation, preventing applying high pressure from the ventilator on the alveoli (plateau pressure) and maintaining patient-ventilatory synchrony.

Some investigators found that patients with ARDS from COVID-19 had preserved lung mechanics (relatively high lung compliance) despite the presence of severe hypoxemia [8]. Contrary to the usual ARDS where severe hypoxemia is associated with poor lung mechanics. They hypothesized that the pathogenesis of COVID-19 ARDS is different from the usual ARDS and suggested a different approach to ARDS management [8]. They proposed two different types of COVID-19 ARDS: (1) an L-type with high respiratory compliance, low recruitability and lower lung weight. (2) An H-type that is similar to the typical ARDS with low respiratory compliance, high recruitability and higher lung weight with extensive consolidation [9]. For the L-type, they suggested modifying ARDS management using higher tidal volume (8-9 m/kg), lower PEEP, no prone positioning except as a rescue maneuver and early intubation to prevent self-inflicted lung injury. The H-type should be treated with higher PEEP, low tidal volume (6 mL/kg), prone positioning [9].

Many patients with COVID-19 ARDS will end up needing mechanical ventilation. These patients have a high CFR that can range from 47% to 84% according to their age [10]. Understanding the lung mechanics in COVID-19 ARDS patients will help our understanding of the pathophysiology of this disease and the search for the best management. This in turn should improve the hospital and ICU outcomes of COVID-19 patients. In this study, we looked into the respiratory pathophysiology of mechanically ventilated COVID-19 patients with acute respiratory distress syndrome at Ohoud Hospital, Madinah, Saudi Arabia. 

## Materials and methods

Type of study

An observational retrospective cohort study was conducted on adult COVID-19 patients who needed mechanical ventilation in Ohoud Hospital, Madinah, KSA from 22.06.2020 to 27.09.2020.

Ethical approval

Ethical approval was obtained from the Medical Research Ethics Committee, Taibah University, Madinah. Approval was also obtained from: Madinah Health Affairs Directorate, Ministry of Health, and Ohoud Hospital, Ministry of Health, Madinah.

Inclusion criteria

· Adult patients aged more than 18 years.

· Acute respiratory distress syndrome from COVID-19.

· Patients who needed mechanical ventilation.

· Day time collected records.

· Records from the day of intubation or 1-2 days after.

· Male and female patients.

· Patients with or without comorbidities

Exclusion criteria

· Missing or insufficient data.

· ARDS not attributed to COVID-19.

Sample size

A total of 92 patients, 40 (43.4%) of them were excluded because they did not meet the inclusion criteria. Fifty-two (56.6%) patients were included.

Place of study

Intensive Care Department at Ohoud Hospital in Madinah, KSA.

Data collection

Patients admitted to the intensive care unit department with a diagnosis of COVID-19 who developed ARDS needing mechanical ventilation were selected as possible candidates for the study. Subsequently, we reviewed their medical charts and electronic medical records to obtain the necessary data.

The collected data included:

Demographic data.

Medical history and comorbidities.

Mechanical ventilation settings and numbers.

Other sources of oxygen used to treat the study patients.

Medications: sedation, respiratory medications, inotropes and diuretics.

The duration of Intensive Care Unit admission, intubation, and number of days on ventilators were recorded.

Statistical analysis

We performed descriptive statistical analysis using Statistical Package for the Social Sciences (SPSS) software package version 22 (IBM Corp., Armonk, NY) to summarize the following data: changes in oxygenation parameters in COVID-19 respiratory failure patients and response to established management for COVID-19 respiratory failure patients.

Other statistical analyses included calculating the driving pressure by subtracting PEEP from Plateau Pressure and calculating the Static Compliance by dividing Tidal Volume over Driving Pressure and calculating the PaO_2_/FiO_2_ ratio.

## Results

In this study, a total of 52 intubated patients with laboratory-confirmed COVID-19 were intubated and admitted to the ICU at Ohud Hospital in Medina, KSA. The patients’ age range was 41-95 years (Table 1). The median age was 63 years (range, 41-92 years), most of the participants were males (33 (63.5%)) and 19 (36.5%) were females (Table 2).

**Table 1:** Patients’ demographics.

	Median	Minimum	Maximum	Mean	SD
Age	63	41	95	64	13.1
Height	165	145	185	166.9	8.8
Weight	61.5	39	79	61.7	9.4

**Table 2:** Gender.

	Frequency	Percent
Female	19	36.5
Male	33	63.5

Comorbidities

Twenty-seven patients (51%) had diabetes, 27 patients (51%) had hypertension, seven patients (13.4%) had bronchial asthma, four patients (7.6%) had ischemic heart disease, three patients (5.7%) had bronchopneumonia, and three patients (5.7%) had chronic kidney disease.

Respiratory parameters on intubation

The Berlin criteria were used to classify ARDS:

 PaO_2_/FiO_2_ ratio ≤300 and >200 is mild ARDS; PaO2/FiO2 ratio 100-200 is moderate ARDS; PaO_2_/FiO_2_ ratio, respiratory system compliance (≤40 mL/cm H_2_O), positive end-expiratory pressure (≥10 cm H2O).

On intubation, 25 patients (76.9%) had severe ARDS according to the Berlin criteria for ARDS (PaO_2_/FiO_2_ ratio< 100), and 14 patients had moderate ARSD (PaO_2_/FiO_2_ ratio 100- 200).

On intubation, the median PEEP was 10 cm H_2_O (IQR, 2.3-16), the median plateau pressure was 27 cm H_2_O (IQR, 12-40), and the median driving pressure was 17 cm H_2_O (IQR, 3-30). The median static compliance of the respiratory system was 24.7 mL/cm H_2_O (IQR, 12.8-153.3) (Table 3).

**Table 3:** Respiratory parameters on intubation. PaO_2_/FiO_2_: partial pressure of arterial oxygen/fractional inspired oxygen; PEEP: positive end-expiratory pressure;* *P Plateau*:* plateau pressure; PIP*:* peak inspiratory pressure.

	N	Range	Median	Minimum	Maximum	Mean	SD
PaO_2_/FiO_2_ ratio	41	179.0	85	25.0	204.0	95.680	43.8837
PEEP	52	13.7	10	2.3	16.0	10.275	2.9015
P plateau	52	28.0	27	12.0	40.0	26.192	5.9013
Driving pressure	52	27.0	17	3.0	30.0	15.917	5.6865
Static compliance	52	140.5	24.7	12.8	153.3	32.621	26.2415
PIP	51	32.0	31	13.0	45.0	29.863	6.8732

ICU therapies

Twenty-seven patients (51.9%) were on non-invasive positive pressure ventilation, and 27 patients (51.9%) were on a high-flow nasal cannula (Table 4).

**Table 4:** Patient characteristics.

All patients		Characteristics
Number of patients	Percentage of patients (N = 52)	
		Demographics
52/52	63 (41-92)	Age, year, median (range)
		Sex, n (%)
33/52	63.5%	Male
19/52	36.5%	Female
51/52	165 (145-185)	Height median (range)
51/52	61.5 (39-79)	Weight median (range)
51/52	22.6 (18.5-23.1)	BMI median (range)
		Comorbidities
27/52	51%	DM
27/52	51%	HTN
7/52	13.4%	Bronchial asthma
4/52	7.6%	Ischemic Heart Disease
3/52	5.7%	Bronchopneumonia
3/52	5.7%	Chronic Kidney Disease
		Respiratory parameters on intubation
41/52	85 (25-204)	PaO_2 _:FIO_2_ , median (IQR)
		Ventilator parameters on intubation, median (IQR)
52/52	10 (2.3-16)	Positive end-expiratory pressure, cm H_2_O
52/52	27 (12-40)	Plateau pressure, cm H_2_O
52/52	17 (3-30)	Driving pressure, cm H_2_O
52/52	24.7 (12.8-153.3)	Static compliance, ml/cm H_2_O

Medications

Sedation

Fifty patients (96.1%) were taking fentanyl, 41 patients (78.8%) were taking propofol, 29 patients (55.7%) were taking atracurium, and 16 patients (30.7%) were taking benzodiazepine (Table 5).

**Table 5:** Patient characteristics (continued).

All patients		Characteristics
Percentage of patients (N = 52)	Number of patients	
		Medications
		Sedation
50/52	96.1%	Fentanyl
41/52	78.8%	Propofol
29/52	55.7%	Atracurium
16/52	30.7%	Benzodiazepine
		Respiratory Medications
9/52	17.3%	Ventolin
5/52	9.6%	long-acting β_2_ agonist
5/52	9.6%	Pulmicort
2/52	3.8%	Atrovent
1/52	1.9%	Inhaled steroid
		Inotropes
34/52	65.3%	Norepinephrine
2/52	3.8%	Dopamine
1/52	1.9%	Epinephrine
1/52	1.9%	Vasopressin
		Diuretics
8/52	15.3%	Diuretics

Respiratory medications

Nine patients (17.3%) were taking Ventolin, five patients (9.6%) were taking long-acting β2 agonist, five patients (9.6%) were taking Pulmicort, two patients (3.8%) were taking Atrovent, and one patient (1.9%) was taking an inhaled steroid (Table 5).

Inotropes

Thirty-four patients (65.3%) were taking norepinephrine, two patients (3.8%) were taking dopamine, one patient (1.9%) was taking epinephrine, and one patient (1.9%) was taking vasopressin (Table 5).

Diuretics

Eight patients (15.3%) were taking diuretics (Table 5).

**Figure 1:**
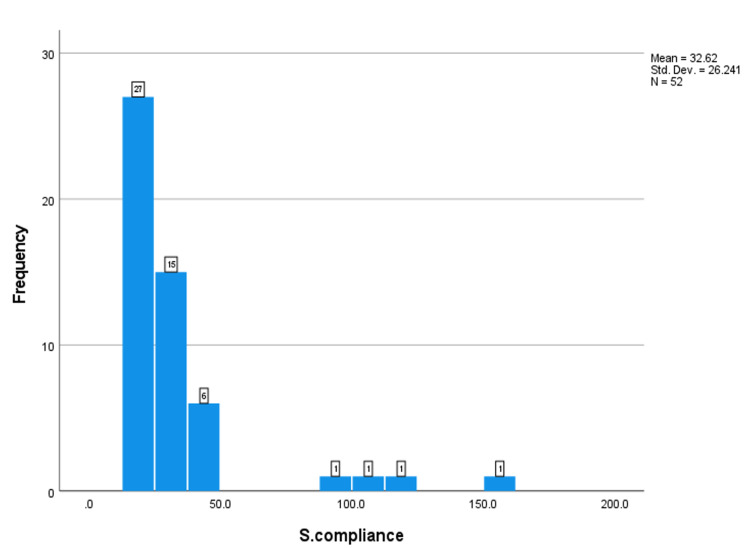
The frequency of the static compliance.

**Figure 2:**
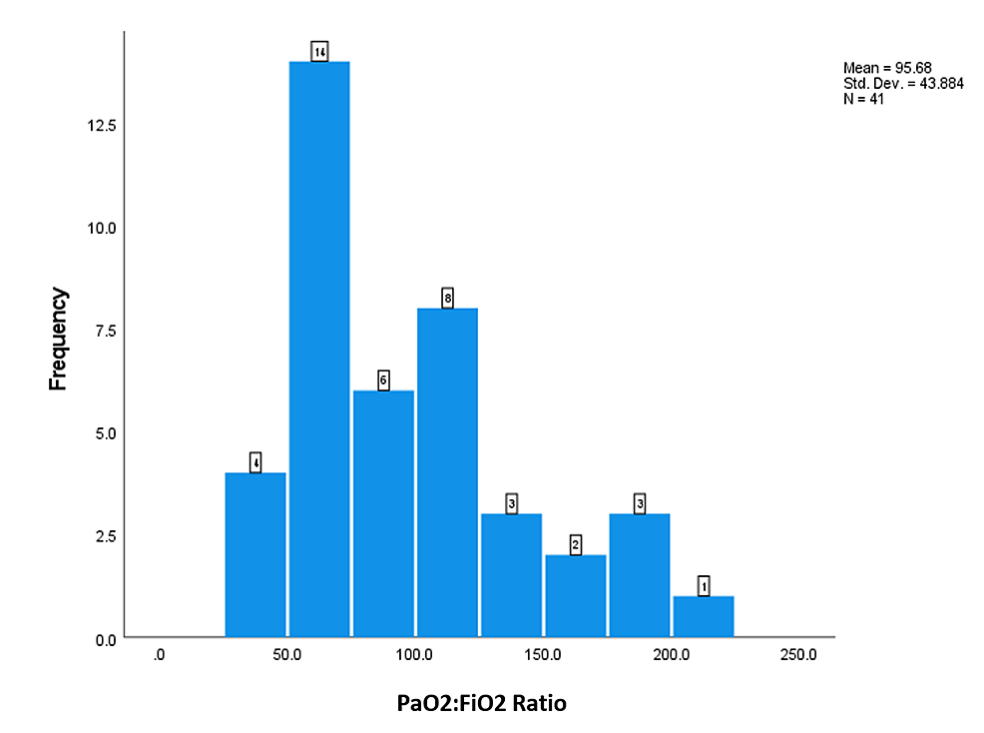
The frequency of the PaO2/FiO2 ratio.

**Figure 3:**
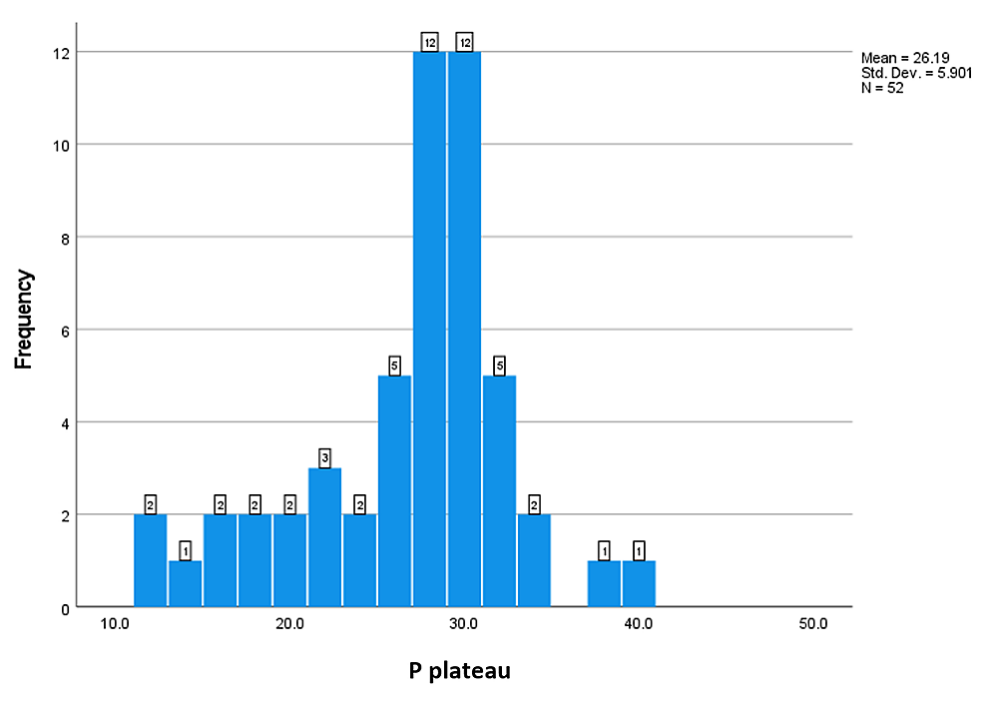
The frequency of the P. plateau.

**Figure 4:**
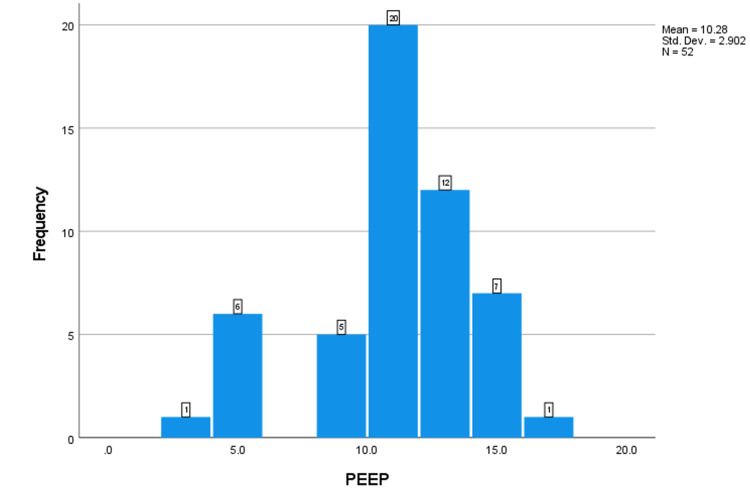
The frequency of the PEEP. PEEP: positive end-expiratory pressure.

Respiratory indices during the first three days after intubation, including the PaO_2_:FiO_2_ ratio, plateau pressure (Pplat), positive end-expiratory pressure (PEEP), and static compliance of the respiratory system (CstatRS), were obtained in intubated patients with coronavirus disease (COVID-19) respiratory failure. The number of patients with recorded values is shown below the Y-axis. The X-axis indicates the median value (Figures 1-4).

## Discussion

This study aimed to investigate the lung mechanics of COVID-19 patients with ARDS managed with mechanical ventilation. The median respiratory system compliance in this cohort of patients was 24.7, which is similar to previously reported cohorts of non-COVID-19 ARDS [8,11]. Only two patients out of 52 had near-normal respiratory system compliance. These results contradict the early studies on COVID-19 ARDS, which reported that many of these patients have preserved lung compliance and lack of lung recruitability [8,11]. This study supports the findings of other studies that found no difference in lung mechanics between COVID-19 and non-COVID-19 ARDS patients [12]. The median PO2:FiO2 ratio in this study was 85 mmHg, which is significantly lower than in early reports on COVID-19 ARDS patients. 76.9% of the patients had severe ARDS according to the Berlin criteria. The median PaO2:FiO2 in Ziehr et al.’s study was 182 and in Schenck et al.’s study, it was 103 [12,13]. This difference can be explained by the fact that early in the pandemic, it was suggested that COVID-19 ARDS patients should be intubated early, which was expected to improve their PaO2:FiO2. Some studies [8,12] intubated almost all hypoxic COVID-19 patients on the first day of ICU admission. Another study recommended against the routine use of a high-flow nasal cannula or non-invasive ventilation and favored immediate invasive mechanical ventilation [12]. In our hospital, we advocated for a trial of high-flow nasal cannula and/or non-invasive ventilation before proceeding to invasive mechanical ventilation. About 51.9% of our cohort of patients had either used high-flow nasal cannula or non-invasive ventilation. Other findings worth mentioning in this cohort of patients is the high percentage of diabetes mellitus and hypertension (51% and 51%, respectively). Another study reported that 26% of their study population had diabetes mellitus and 44% had hypertension. Hypertension and DM have been associated with increased risk of severe COVID-19 infection and ARDS [14,15].

Moreover, although the average BMI of patients in our sample was ideal (18.5-23.1), they showed signs of severe ARDS. Thus, we cannot conclude that a higher BMI is associated with more severe ARDS in COVID-19 patients.

The limitations in our study include small sample size, limited duration of follow-up, and one hospital-based sample.

## Conclusions

Our study found that patients with COVID-19 and ARDS who needed mechanical ventilation had the same lung mechanics when compared to a cohort of patients with non-COVID-19 ARDS. We observed a lower PaO_2_:FiO_2_ ratio in these patients after mechanical ventilation. This can be attributed to tolerating a lower level of hypoxia and a trial of high-flow nasal cannula and/or non-invasive ventilation before initiating invasive mechanical ventilation. We need more studies to describe the biological and unique features of COVID-19, which would help clarify the best management of COVID-19 ARDS. Until then, we suggest treating ARDS in COVID-19 patients using conventional ARDS management strategies.

.
